# Model to support intervention prioritization for the control of *Aedesaegypti* in Brazil: a multi-criteria approach

**DOI:** 10.1186/s12889-022-13006-1

**Published:** 2022-05-10

**Authors:** Lucas A. dos Santos, Ana Flávia A. dos Santos, Amanda G. de Assis, João F. da Costa Júnior, Ricardo P. de Souza

**Affiliations:** 1 Centro de Tecnologia, Universidade Federal do Rio Grande do Norte, 59072-970, Natal, Brazil; 2 Centro de Ciências Aplicadas, Universidade Federal do Rio Grande do Norte, 59072-970 Natal, Brazil

**Keywords:** Multicriteria decision analysis, MCDA, Epidemiological surveillance, Arbovirus control

## Abstract

**Background:**

Despite continuous strategic investments to mitigate the complexity involving arboviruses control, it is still necessary to further research methods and techniques to achieve in depth knowledge and shorter response times in the application of intervention activities. Consequently, the current work focused its efforts on the development of a multicriteria decision support model for the prioritization of prompt response activities for *Aedes aegypti* control, based on a case study in the city of Natal/RN.

**Method:**

The research was carried out in three stages: a) preliminary; b) modelling and choice; and c) finalization; the second stage was made possible by the Flexible and Interactive Tradeoff (FITradeoff) method for ranking problematic. Furthermore, the research encompassed ten actors who were involved in the model construction, eight internal and two external to the Natal Zoonoses Control Center (ZCC-Natal) as well as the observation of four operating scenarios for arboviruses control, based on transmission levels; and, evaluation of eleven alternatives from six different criteria perspectives.

**Results:**

Rankings of the interventions evaluated in each of the four control operation scenarios present in the city of Natal/RN were obtained, considering technical criteria guided by the Pan American Health Organization (PAHO).

**Conclusions:**

As a result, it was developed a structured decision-making model that could help decision makers to minimize the effects and risks associated with the proliferation of the vector.

## Background

In order to address the inherent complexity of health decisions derived from factors such as uncertainty, conflicts of interest and subjectivity in the assessment of health technologies, amongst other aspects [1, 2], the multi-criteria decision analysis (MCDA) emerges as a structured methodology to support the decision-making processes, differing from other formal approaches due to its characteristic of mathematically modelling the subjectivity present in the f decision-makers’ judgement [3]. Hence, this methodological approach analytically guides decision support in a rational and transparent manner by aiming at the best choice in face of a set of alternatives, based on the decision maker’s preferences [4, 5].

Although the application potential of MCDA is evidenced in different areas such as health technology assessment, support to the selection of screening protocols and patient admission processes [4, 6, 7], there was a gap in the context of infectious diseases, like those transmitted by the *Ae aegypti* vector, given that the scientific productions that explore MCDA and tropical diseases focus on supporting the identification and risk areas spatial classification [8], mainly ignoring research opportunities to support the prioritization of interventions for the vector control, a process without consensus on the systematization of actions, despite the general guidelines recommended by the Pan American Health Organization (PAHO) [9].

In Brazil, the recurring mobilization for the control of arboviruses, especially those transmitted by *Ae aegypti*, is a testament to the great risks that the viruses transmitted by this vector have been causing to human health; with the proliferation of diseases such as Dengue, Zika and chikungunya; *Ae aegypti* has been the main cause for deaths by infectious diseases across the country [10, 11].

Mechanisms designed to mitigate the proliferation of these diseases are prioritized by public entities, the 16-year Bulletin (2003-2019) developed by the Health Surveillance Secretariat of the Ministry of Health presented the need for applied research to be considered as a strategic investment in a control intervention project [12].

The multi-criteria approach is flexible as well as capable of taking into account multiple factors considered important for public policy managers, for instance, the case in which the interventions applied are sustained for a pre-defined period of time without harming their overall performance [13]. The proper management of these and other factors are essential to the development of appropriate methods given that the action taken may not meet expectations due to inadequate planning [14], which justifies the need for a formal procedure that, at the same time, time that meets the general aspects recommended by PAHO, is adapted to the specificities of the analysed region.

Thus, the current work utilizes MCDA concepts to develop a multi-criteria model that supports the decision-making process for prioritizing *Ae aegypti* control interventions by a public health organization in the Northeast.

Four Operational Scenarios (OSs) are considered which can be distinguished by the risk severity of disease transmission. The expert evaluations, characteristics of operational scenarios and actions availability was observed to ensure that the means of action are adequate to the prompt response required by each OS.

Flexible and Interactive Tradeoff (FITradeoff) was the method utilized to build the model. The main contribution of the current work lies in the improvement of the use of the multi-criteria approach for health management and public policy, emphasizing disease vectors control, thus helping public policy managers not only in the environment in which the case study was conducted, but also in other geographic regions with similar climatic conditions, which could benefit from the support of a partial information method that requires less cognitive effort from the decision maker whilst preserving the robustness of the tradeoff elicitation procedure.

### Multicriteria decision analysis

The multicriteria decision analysis, unlike other types of analysis, makes explicit a set of logical criteria that will serve as an accessible, accepted and exhaustive communication source, allowing for a thorough comprehension about preference shifts within the decision-making process [15]. Its potential to support the decision-making process is far superior than other traditional methods, as it enables complex decisions that include multiple criteria and simultaneously consider quantitative and qualitative data, in addition to involving multiple stakeholders [16].

Although all multi-criteria methods share the idea of systematic evaluation by decomposing the general alternative analysis into multiple criteria, there are several types of techniques, each associated with the structure of the decision problem [17].

[15] identifies four reference problems as follows: :

- Choice or selection (problematic *P*.*α*) - amongst a set of actions, a subset is chosen to clarify the decision. This subset comprises “optimal” or “satisfactory” actions;
- Sorting (problematic *P*.*β*) - each action is grouped into categories of specific characteristics;
- Ranking (problematic *P*.*γ*) - realization of an arrangement with the regrouping of actions in an orderly manner;
- Description (problematic *P*.*δ*) - through the description of actions and their consequences, the decision is clarified in term.

In this article, the problematic *P*.*γ* was utilised, which, according to [18], allows for the allocation of alternatives in order of increasing preference, based on the preference model. So that, in the spaces of actions under study, the construction of the ordered list of interventions that best applies to each of the operational scenarios analyzed will be carried out, allowing the comparison between all actions that make up the set of optimal alternatives.

### FITradeoff method for ranking problematic

FITradeoff is composed by a group of segmented multicriteria methods in the utilization of the additive aggregation of preferences in Multi-Attribute Value Theory (MAVT). It was developed by the Centre for Development in Information and Decision Systems (CDSID) of the Federal University of Pernambuco (UFPE), under the coordination of Prof. Dr Adiel Teixeira de Almeida [19].

The method is distinguished in its category by overcoming frictions and inconsistencies commonly seen in the process of eliciting preferences by traditional Tradeoff, since it is motivated by flexible elicitation [20]. That is, it makes use of the traditional Tradeoff axiomatic structure and improves the execution of its procedures by directing cognitively easier questions to the DM, as it works only with strict preference statements; and assume partial or inaccurate information at the beginning of the elicitation process [18, 20].

For the ranking problem, FITradeoff seeks to arrange the alternatives in order of preference based on the understanding of the dominance relationships provided by Linear Programming Problems (LPP), according to the model described below [18].

$$max D(A_{i},A_{k})=\sum_{j=1}^{m}w_{j}v_{j}(A_{i}) - \sum_{j=1}^{m}w_{j}v_{j}(A_{k}) ~~~~~~(1)$$

s.t.

$$w_{1}> w_{2}> \cdots > w_{m}|\sum_{j=1}^{m}w_{j}=1 ~~~~~~~~~~~~~~~~~~~~~~~~~(2)$$

$$w_{j}v_{j}\left (x_{j}^{'} \right)> w_{j+1} ~~~ j=1 ~~~ to ~~ m-1 ~~~~~~~~~~~~~~~~~~(3)$$

$$w_{j}v_{j}\left (x_{j}^{\prime\prime} \right)< w_{j+1} ~~~ j=1 ~~~ to ~~ m-1 ~~~~~~~~~~~~~~~~~~(4)$$

$$w_{j}\geq 0 ~~~j=1,\cdots, m ~~~~~~~~~~~~~~~~~~~~~~~~~~~~~~~~~~~~~~~(5)$$

The model seeks to maximize through the function *MaxD*(*A*_*i*_,*A*_*k*_) the global value (*w*_*j*_*v*_*j*_[*A*]) of the difference between each pair of alternatives (*A*_*i*_,*A*_*k*_) within a considered weight space. This weight space is built from the ranking of the scale constants (*w*_*i*_), according to (2) and superior limits definition (3) as well as inferior (4) presumed in each criterion. Furthermore, in (2) there is the normalization; and (5) the non-negativity of weights [18]. It is worth noting that, despite the term “weight” being used in the description of the variables w, these refer to scale constants and do not exclusively determine the degree of importance of each criterion.

Finally, after the operationalization of the LLPs, for each pair of alternatives, a matrix with the dominance relationships is provided, allowing the construction of the partial or complete alternatives ranking [18].

## Methods

The Zoonoses Control Centre (ZCC) was defined in the city of Natal, capital of Rio Grande do Norte as the development field for the case study, given that it is the agency responsible for the operationalization of vector control in the locality.

As for the selection of interventions for vector control, the ZCC has worked with the verification of priority areas, allocated in operational scenarios, allowing for a faster understanding of risk levels. Currently, the study unit is segmented into four different OPSs, as follows:

- Operational Scenario 1 (OS 1) - areas that have a stable egg density diagram below the median, presence of traps with stable egg density index of *aedes sp.*, no persistent traps and no notification of reports;
- Operational Scenario 2 (OS 2) - areas that present an unstable egg density diagram, presence of traps with unstable egg density index of *aedes sp.*, presence of vector productivity with unstable egg density index, reports registered and unstable case incidence diagram;
- Operational Scenario 3 (OS 3) – areas with egg density diagram in progression above the maximum threshold, presence of traps with *aedes sp.* egg density index in progression above the median, presence of vector productivity with egg density index of *aedes sp.* in progression, diagram of incidence of cases in progression above the median, reports of epizootics suggestive of arboviruses, increase in the rate of wild vectors for transmission of yellow fever or other atypical arboviruses in urban areas;
- Operational Scenario 4 (OS 4) - Diagram of egg density in progression above the maximum threshold presence of trap with egg density index of *aedes sp.* in progression above the maximum threshold, presence of vector productivity with egg density index of *aedes sp.* in progression, diagram of the incidence of cases in progression above the maximum threshold, record of epizootics suggestive of arboviruses, presence of wild vectors for transmission of yellow fever or other atypical arboviruses in urban areas.

The model developed in the current article will provide support for each of the scenarios, considering their specific characteristics as well as activities that present an optimal potential for risk control. Therefore, it is structured according to the three phases shown in the framework [21] described in Table 1, namely: preliminary phase, modelling and choice phase, and finalization phase; each of them composed of specific steps that will be discussed next.

**Table 1:** Problem structuring framework

Preliminary Stage	Modelling Stage	Finalization Stage
1. Characterize decision maker(s) and other actors;	6. Preferences modelling;	9. Evaluate alternatives;
2. Identify goals;	7. Intra-criterion evaluation;	10. Perform Sensitivity analysis;
3. Establish criteria;	8. Inter-criteria evaluation;	11. Analyse results and suggest;
4. Establish the problem and alternatives;		12. Implementing the decision.
5. Identify uncontrolled factors;		

### Preliminary stage

Four types of actors were identified as well as their respective roles in the model construction process, namely: i) the Decision Maker (Director of ZCC-Natal) with the role of evaluating the available information about the problem and declare preferences in the elicitation process, being responsible for the decisions made by the unit; ii) the General Specialists (Technical Manager and Field Operation on Duty) with the role of assisting in the evaluation of general information collected in the internal units; iii) the Technical Specialists (Heads of the Epidemiological, Entomological, Geostatistical, Education and Finance centres, and Field Operation Supervisor) responsible for informing variables related to the problem analysed from a daily actions perspective in each sectorial unit; iv) the Analysts (the current authors), who were given the responsibility to mediate the MCDA methodological process and build the model.

The unit’s guiding objectives for the selection process of interventions for vector control were summarized as follows:

I: Improve the level of involvement of the population and impact results on the disease control;
II: Decrease the costs associated with the actions, whether in the process of purchasing materials, employment costs, rental costs and so forth;
III: Enhance the control tool’s ability to reach the expected effect;
IV: Decrease the need to establish new applications;
V: Increase the community’s trust and acceptance regarding the interventions;
VI: Organizing actions that have a proactive and sustainable impact on the combat against the epidemics;

The criteria establishment stage encompassed the criteria associated with each of the identified objectives, considering their nature and measurement units as shown in Table 2.

**Table 2:** Characterization of the evaluated criteria

Criteria	Description/Scale	Function
(ALI) Application level and impact;	Level of involvement of the population and the results of impact on the disease: (1) Individual, (2) Household, (3) Neighbourhood or (4) District-local;	Maximize
(C) Cost	Estimate of the amount invested in resources from acquisition to application of the action, evaluated at 5 level: from (1) very low to (5) very high;	Minimize
(E) Effectiveness	Ability of the control tool to achieve the expected effect, evaluated at 5 level: from (1) very low to (5) very high;	Maximize
(R) Reapplication	Need to establish new applications in the same area in a short period of time, measured in a binary way: (1) yes or (0) no;	Minimize
(SA) Social Acceptance	Level of trust and acceptance of the intervention by the community, evaluated at 5 levels: from (1) very low to (5) very high;	Maximize
(S) Sustainability	Efficacy of the tool to maintain the vector population at sustainably low levels, measured in a binary way: (1) yes or (0) no;	Maximize

The current authors chose to address the “cost” criterion with the respective assessment scale in verbal terms, based on guiding intervals as shown in Table 3. Such intervals were constructed through the cost estimate associated with the operationalization of each alternative, from the information provided by the finance unit manager.

**Table 3:** Cost scale parameters

Guidance Range	Scale	Meaning
≤ R$ 2.500,00	1	Very low
>R$ 2.500,00 ≤ R$ 5.000,00	2	Low
>R$ 5.000,00 ≤ R$ 7.500,00	3	Medium
>R$ 7.500,00 ≤ R$ 10.000,00	4	High
>R$ 10.000,00	5	Very

The alternatives refer to the set of actions available to the decision maker [22]. Thus, 11 alternatives that constitute the list of unit-specific solutions for vector control were considered:

(A) Residual application of adulticides;

(B) ULV cold fogging - heavy duty;

(C) ULV cold fogging - portable;

(D) Press Call;

(E) Biological larvicides;

(F) Door-to-door orientation;

(G) Mechanical removal from breeding sites;

(H) Educational campaigns;

(I) Population alert walk;

(J) School campaigns;

(K) Lectures and discussion groups;

The problem considered to guide decision support as well as the model’s recommendations was that of (*P*.*γ*) ranking. The choice was deemed as adequate by the actors since the purpose of (*P*.*γ*) is to propose an arrangement of actions by regrouping them in order of preference [15]. The construction of an orderly ranking of interventions is planned in the present study in order to determine those that best meet the decision-maker’s preferences within each operating scenario.

Factors that were not controlled, such as: cuts or increase in financial investments as well as implementation of new interventions were disregarded as they were not under the control of the decision maker and, consequently, could not be considered in the model.

#### Modelling stage

(P,I) type evaluations will be used in the preference structure, given the DM’s ability to compare all action alternatives in relation to their preferences and indifferences, due to their familiarity with the direction of each of the interventions listed, thus not requiring other types of judgment.

As a result of this, it was decided to follow this type of structure in which preferences will be easily declared and indifferences can be understood in a flexible way, without demanding high DM cognitive efforts. As for rationality, a compensatory approach by the decision maker was identified in some comparisons between alternatives.

Therefore, having identified the adequacy to the structure (P,I), the need to reduce efforts in defining indifferences and the use of compensatory rationality, the FITradeoff multicriteria method showed its application potential, being chosen as a potential solution for the problem analysed.

Once the application method was defined, phase 7 (intra-criterion evaluation), 8 (inter-criteria evaluation) and 9 (alternatives evaluation) were carried out simultaneously, with the development of preference elicitations, enabled by the computer system.

### Finalization

Sensitivity Analysis makes possible to verify whether preliminary findings are vulnerable to changes in the model, generally verified from three perspectives: i) technical - with changes in model parameters; ii) individual - with the assessment of the decision maker’s comfort regarding the results, through her intuition and understanding of the problem; iii) group - capturing the views of different actors [23].

Despite the use of the FITradeoff method fostering greater consistency in the final results with the understanding of rankings in weight ranges for each criterion, in which the ranking of solutions remains unchanged, instead of defining exact values for the scale constants, it was still necessary the sensitivity analysis of the results in face of variations in the model parameters, which is performed technically, with a variation of ± 10 in input performances; and individual assessment by the ZCC-Natal/RN manager.

At this stage, the model results were compared with the existing practices in the study unit, in order to observe convergences and/or divergences between the two perspectives: model versus standard practices.

## Results

**Fig. 1:**
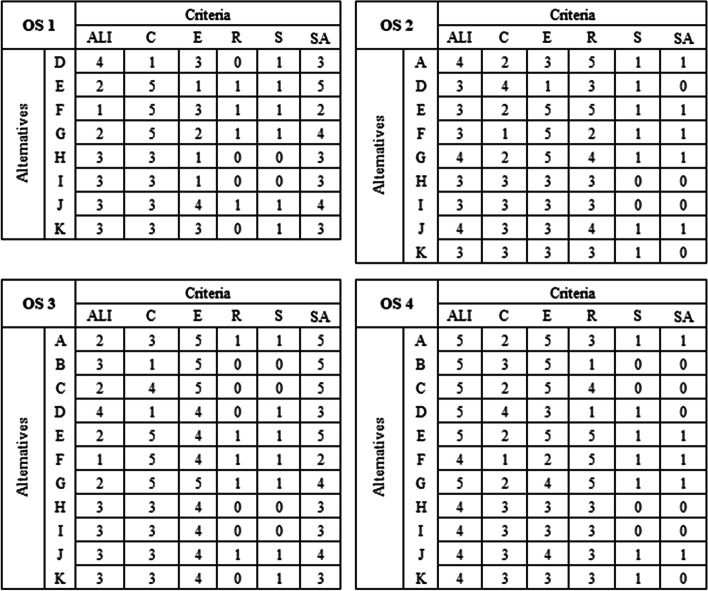
Consequence Matrix by Operational Scenarios

For each of the Operational Scenarios, a matrix of consequences was built that represents the performance of the alternatives in each of the evaluated criteria. The use of individualized matrices (Fig. 1) was due to the variation in the number of alternatives applicable to each OS and the variations in the effectiveness analysis in the scenarios, given that an action or decision does not have the same effectiveness in all application conditions.

Therefore, the ranking of the scale constants for each scenario were carried out, through the systematic evaluation, followed by the elicitation cycles, procedures that allowed the construction of the final panorama of interactions presented in Table 4.

**Table 4:** Summary of interactions in the operating system

Operation Scenario	Ranking of Scales	Number of cycles	
1	*w*_*E*_>*w*_*ALI*_>*w*_*SA*_>*w*_*C*_>*w*_*R*_>*w*_*S*_	7	
2	*w*_*E*_>*w*_*ALI*_>*w*_*C*_>*w*_*SA*_>*w*_*S*_>*w*_*R*_	7	
3	*w*_*E*_>*w*_*SA*_>*w*_*C*_>*w*_*ALI*_>*w*_*S*_>*w*_*R*_	10	
4	*w*_*E*_>*w*_*ALI*_>*w*_*SA*_>*w*_*C*_>*w*_*S*_>*w*_*R*_	8	

**Fig. 2:**
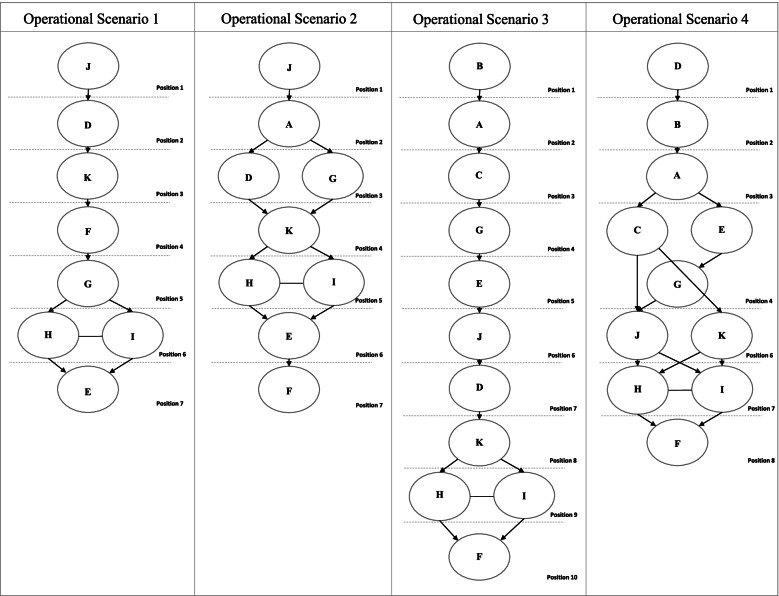
Ranking of interventions by scenarios and preferences

Thus, the development of the final ranking of alternatives was carried out, with the identification of predominant relationships, indifferences and incomparability between the evaluated actions. A Hasse Diagram outlines for each OS (Fig. 2) the relationship between the alternatives, observing the ranking of the scale constants and the decision-maker’s preference statements.

The diagrams in Fig. 2 display the dominance relationships between each of the alternatives. The ellipses represent the problem’s alternatives; the directed arrows indicate the dominance relationship; solid lines between ellipses indicate indifference between alternatives and dotted lines indicate ranking positions.

**Fig. 3:**
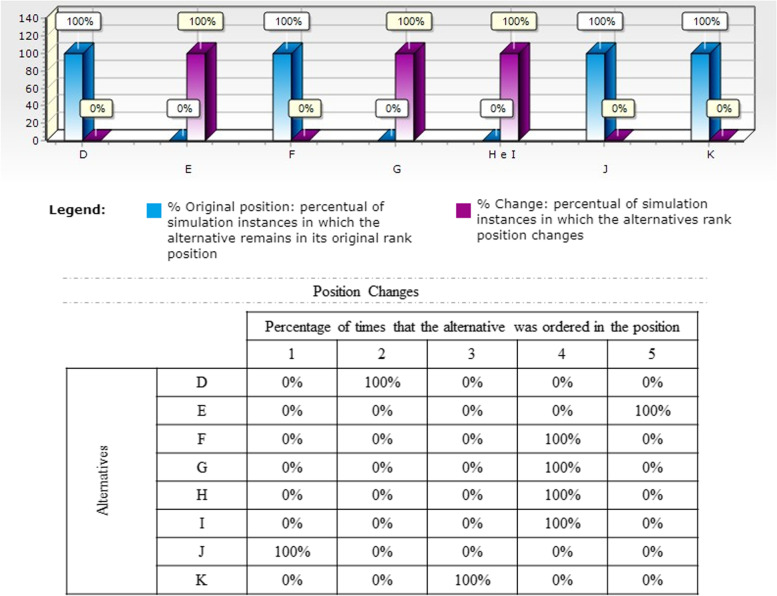
Model Sensitivity Analysis - OS 1

**Fig. 4:**
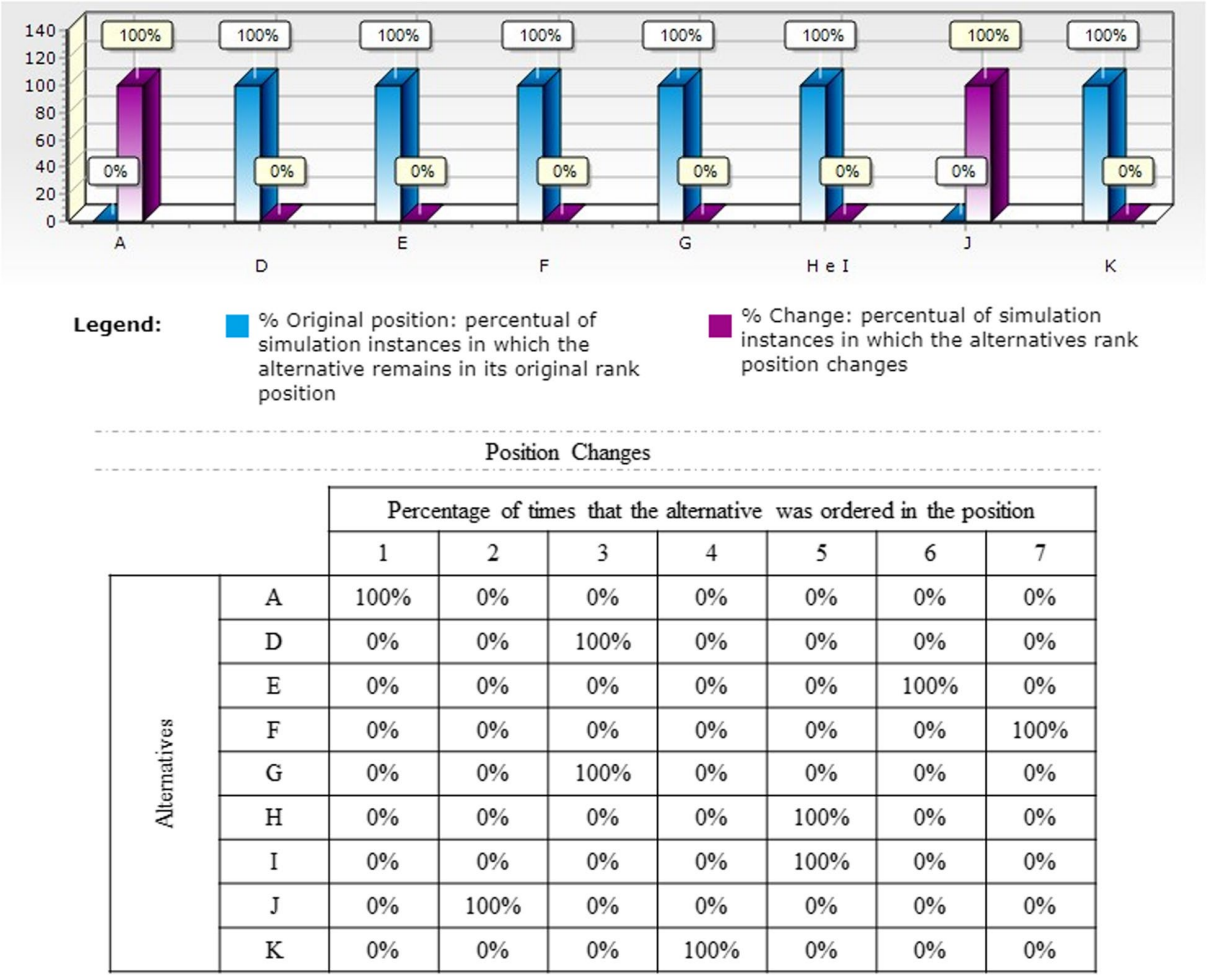
Model Sensitivity Analysis - OS 2

**Fig. 5:**
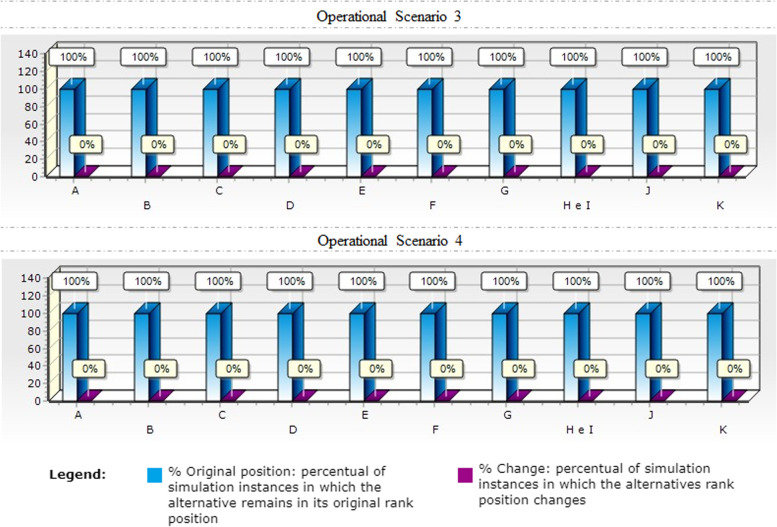
Model Sensitivity Analysis - OS 3 and 4

Once the ordination was defined, the robustness of the model of each OS was measured through technical sensitivity analysis, performed to assess the variability of the recommendations against possible changes in the performance of interventions in ± 10 within all criteria. Results are analysed according to how often the alternatives remained in their nominal positions (Figs. 3, 4, 5).

With the OS 1 sensitivity analysis, Fig. 3, a reduction from 7 to 5 positions in the ranking was verified in 100 of the simulated scenarios. As for the positioning of the alternatives, the first four (J, D, K and F) remained fixed in all analysis cycles. The last four (G, H, I and E) were displaced to a higher position due to the repositioning of alternatives G, H and I in position 4, which consequently pushed the last activity to positions 5. This variation can be justified by the proximity of the performances that the latter alternatives have in the low transmission scenario.

As for OS2, Fig. 4, only the first two alternatives (A and J) showed position changes, and in 100 of the simulations. However, this change was driven by the exchange between alternatives A and J, with the number of positions and allocation of the other 7 alternatives unchanged.

Finally, in scenarios 3 and 4 the original position remained unaffected, with no change in ranking, in 100 of the simulation scenarios as shown in Fig. 5.

Thus, the technical sensitivity analysis indicated the robustness of the model, with an opening to small rearrangements in lower risk scenarios, depending on the decision-maker’s judgment, and a more consolidated structure in higher risk scenarios.

## Discussion

The decision-making context found in the current work was highly complex due to the instability of its dimensions, mainly because its processes were influenced by different government spheres. However, the model, as defined by Roy [15] and Almeida [19], achieved its objective by designing in a simplified manner the decision environment faced for the prioritization of interventions for the control of *Ae aegypti*.

As for the findings, as proposed in the article objectives, it was possible to understand the main dimensions that make up the control of arboviruses transmitted by *Ae aegypti*, together with the ecosystem of organizations that direct mitigation efforts; as well as prioritization by ranking interventions in each of the operational scenarios. This perspective lends a degree of flexibility to the prioritization process by providing a set of potentially optimal alternatives.

Thus, the stratification of the action areas based on transmission patterns was supported with the definitions of more efficient interventions, seeking to support the interruption of the vector’s life cycle, based on duly measurable and corroborating criteria with the control units’ objectives.

However, it is important to notice that the model was established based on its controlled factors, disregarding the elements which are out of control. Even with such consideration, important observations were highlighted, with the fact that, in these circumstances, there is a tendency to prioritize the greatest scope of performance for the criteria “effectiveness” and “Level of application and impact”, because despite the need to act on the environment in order to maintain vector stability, it is necessary to think of actions that encompass a larger area with the resources invested, such as: human resources. Thus, the set of actions can be assertive to stabilize and/or positively impact the minimization of the scenario.

As for the ordinations, in the evaluation process of scenario 1, with mild and highly controlled characteristics, activities at the level of orientation to prevention stood out, with those of an educational nature that have the potential to propagate positive results for a longer period and in a large part of society in the first positions.

For scenario 2, the environment undergoes an evolution of the vector’s performance with the verification of instability in the egg density index and record of case reports, generating imminent aggravations. Thus, the formation of brigades is still highlighted as it brings very significant results to the scenario, followed by the residual application of adulticides, as the instabilities have already been detected and it is necessary to break the transmission chain by eliminating the adult vectors that can spread the diseases. However, it is important to note that at level 3 there was the press call as well as mechanical removal of breeding sites because it is a time when it is essential to encourage the involvement of the population to become agents of removal from their homes, as this activity can impact in breaking the vector’s proliferation flow. And, finally, the door-to-door orientation, because it demands a high involvement of resources (both agents and execution time).

In OSs 3 and 4, as they are latent risk scenarios due to the high incidence of vectors, combat interventions that generate immediate results were favoured. Education activities, on the other hand, ranked lower because they are less decisive, as they have long-term results.

Thus, the ranking of interventions for each considered operative scenario allowed for an understanding of the likely results from each of their applications. It is also necessary to emphasize that the proposed model presents the activities recommended in each OS according to the endemic panorama of the city of Natal/RN, which does not guarantee that for all other locations that control the *Ae aegypti* vector, these activities have the same performance and relevance. However, it still becomes a guiding parameter to understand the variables and behaviours patterns associated with the process.

## Conclusions

The MCDA approach has enabled new ways to solve complex problems in different sectors. With regards to Brazilian public health, the multicriteria analysis exposed its potential to allocate efforts to mitigate the negative impacts caused by *Ae aegypti*, a vector that enables constant risks to human health.

The employment of procedures to develop a model to support the decision-making process for prioritizing vector control interventions resulted in a structured decision-making model that can help decision makers to minimize the effects and risks associated with the proliferation of the *Ae aegypti* vector, based on robust and accurate information, as expressed in the model sensitivity analysis.

By focusing on an approach that is still unrelated to the area, the current study is encouraging the search for knowledge and practices related to MCDA methodologies applied to arboviruses control. It is worth noticing, however, that the current research was limited to specific characteristics of the field of study, considering the scenarios and alternatives available in the organization studied.

As for future research, it is valid to work on the decision context under a new perspective: the classification, seeking to group the alternatives into specific levels of intervention to choose sets that integrate techniques of different natures (chemical, biological, mechanics, etc.), as directed by the Pan American Health Organization. Finally, since modelling real processes is a challenge, given its diverse requirements as well as the conflicts of interest between stakeholders, the authors suggest the aggregation of other methodologies that facilitate the development of the model, such as the use of social science theories in the preliminary stage. The aggregation of Activity Theory, for instance, will contribute to the stage of identification and characterization of decision makers and other actors, and consolidation of local social constraints that influence the problem, ensuring the success and greater social acceptance of the proposed model [24].

## Data Availability

All data generated or analysed during this study are included in this published article [and its supplementary information files].
